# Semi-Rational Design of L-Isoleucine Dioxygenase Generated Its Activity for Aromatic Amino Acid Hydroxylation

**DOI:** 10.3390/molecules28093750

**Published:** 2023-04-27

**Authors:** Jianhong An, Jiaojiao Guan, Yao Nie

**Affiliations:** 1 School of Biotechnology and Key Laboratory of Industrial Biotechnology, Ministry of Education, Jiangnan University, 1800 Lihu Road, Wuxi 214122, China; ajhbio1978@126.com (J.A.); 15906191696@139.com (J.G.); 2 International Joint Research Laboratory for Brewing Microbiology and Applied Enzymology, Jiangnan University, 1800 Lihu Road, Wuxi 214122, China; 3 Eye Hospital, Wenzhou Medical University, 270 Xueyuan Road, Wenzhou 325000, China

**Keywords:** Fe (II)/α-KG-dependent dioxygenases, L-isoleucine dioxygenase, aromatic amino acid

## Abstract

Fe (II)-and 2-ketoglutarate-dependent dioxygenases (Fe (II)/α-KG DOs) have been applied to catalyze hydroxylation of amino acids. However, the Fe (II)/α-KG DOs that have been developed and characterized are not sufficient. L-isoleucine dioxygenase (IDO) is an Fe (II)/α-KG DO that specifically catalyzes the formation of 4-hydroxyisoleucine (4-HIL) from L-isoleucine (L-Ile) and exhibits a substrate specificity toward L-aliphatic amino acids. To expand the substrate spectrum of IDO toward aromatic amino acids, in this study, we analyzed the regularity of the substrate spectrum of IDO using molecular dynamics (MD) simulation and found that the distance between Fe^2+^, C2 of α-KG and amino acid chain’s C4 may be critical for regulating the substrate specificity of the enzyme. The mutation sites (Y143, S153 and R227) were also subjected to single point saturation mutations based on polarity pockets and residue free energy contributions. It was found that Y143D, Y143I and S153A mutants exhibited catalytic L-phenylalanine activity, while Y143I, S153A, S153Q and S153Y exhibited catalytic L-homophenylalanine activity. Consequently, this study extended the substrate spectrum of IDO with aromatic amino acids and enhanced its application property.

## 1. Introduction

Hydroxyamino acids are unusual amino acids that are commonly found in nature. They exist as secondary metabolites and components of peptides and proteins. Many hydroxyamino acids are components of sugars, peptides and antibiotics and also play an important role in the chemical synthesis of other compounds as precursors and chiral auxiliaries [1,2,3,4,5,6].

Fe (II)- and 2-ketoglutarate-dependent dioxygenases (Fe (II)/α-KG DOs) are a diverse superfamily of proteins that catalyze a variety of oxidative transformations. Industrially, these enzymes are mainly used in the biosynthesis of hydroxylated amino acids and antibiotics [7,8,9,10,11]. Currently, there is a common understanding of the Fe (II)/α-KG DO reaction mechanism for hydroxylation activity. The Fe (II)/α-KG DO requires Fe (II), usually bound by three residues, to form a highly conserved HXD/E…H triad [12]. α-KG forms complexes with iron in a bidentate manner via its C-1 carboxylate and ketone oxygens [13]. When the substrate binds to the enzyme active site (not, however, to the Fe ion), Fe (II)-bound water is displaced, thus creating a site for binding an O_2_ molecules, generating an Fe (III)-superoxo intermediate. The distal oxygen atom of the Fe (III)-superoxo intermediate attacks C2 of α-KG to release CO_2_ and to yield Fe (IV)=O (ferryl) intermediate. The Fe (IV)=O intermediate attacks the C-H closer to the substrate to form a hydroxylation reaction. Therefore, the distance between the iron ion and the C-H of the substrate is very important [13]. However, the regulation of enzyme selectivity of the Fe (II)/α-KG DO-mediated hydroxylation remains unclear. In addition, not enough Fe (II)/α-KG DOs have been developed and identified, and only a few specific hydroxylases have been reported, such as L-proline 3-hydroxylase from *Streptomyces*, L-proline 3-hydroxylase from *Bacillus thuringiensis*, L-proline cis-4-hydroxylase from *Mesorhizobium loti*, L-proline cis-4-hydroxylase from *Sinorhizobium meliloti*, L-proline trans-4-hydroxylase from *Dactylosporangium*, L-proline trans-4-hydroxylase from *Amycolatopsis* and L-isoleucine dioxygenase (IDO) from *Bacillus thuringiensis* [14]. In short, these factors prevent the application of these enzymes in the production of various hydroxylated amino acids. 

IDO is an Fe(II)/α-KG DO that specifically catalyzes the generation of 4-hydroxyisoleucine (4-HIL) from L-isoleucine (L-Ile) [15,16]. In addition to L-Ile as the target substrate, IDO can also act on other L-aliphatic amino acids and has a substrate spectrum toward such amino acid substrates [17,18,19]. We have previously solved the IDO structure and analyzed the catalytic orientation mechanism of its hydroxylation [20].

To further expand the substrate spectrum of IDO reactions, especially toward aromatic amino acids, we used molecular docking and molecular dynamics (MD) to simulate the enzyme dynamics of Fe^2+^ and α-KG, L-amino acids [L-Ile, D-isoleucine (D-Ile), L-leucine (L-Leu), L-norleucine (L-Nle), L-norvaline (L-Nva), L-phenylalanine (PHE) and L-homophenylalanine (HPHE)]. Then, single point saturation mutagenesis at selected mutation sites was carried out based on polarity pockets and residue free energy contribution, thereby expanding the IDO substrate spectrum for aromatic amino acids to enhance their application properties.

## 2. Results and Discussion

### 2.1. MD Simulation of IDO with L-Aliphatic Amino Acids

**Figure 1:**
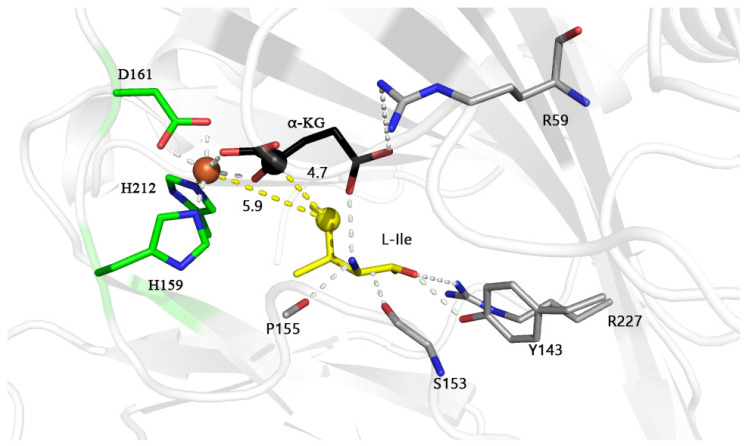
Final structure of the active site of L-isoleucine dioxygenase (IDO) with L-isoleucine (L-Ile) model during molecular dynamics at 200 ns; Fe^2+^, C2 atom of α-KG and C4 atom of L-Ile are shown in brown, black and yellow spheres, and distance between atoms is also indicated by yellow dashed lines. Possible hydrogen bonds are indicated by white dashed lines. The unit of distance is Å.

Our previous structural analysis showed that the IDO monomer (PDB: 6lnh, Chain A) has 240 amino acids (with the N-terminal purification tag removed) and contains a conserved motif H^159^-X_1_-D^161^-X_50_-H^212^ that chelates Fe^2+^, the active site of IDO [20]. Molecular dynamics simulations using Fe^2+^, α-KG and L-Ile as substrates showed that in the IDO active pockets, S153 and P155 formed hydrogen bonds with the amino group of L-Ile, and residues Y143 and R227 had hydrogen bonds to the carboxyl group of L-Ile (Figure 1). Residues S153, P155, Y143 and R227 formed a polar substrate binding pocket, and the formation of this pocket restricted the other end of L-Ile toward Fe^2+^ and α-KG, when the carbonyl group of α-KG (opposite D161 bound to Fe^2+^) and the C1 position carboxyl group (opposite H159, H212 bound to Fe^2+^) are bound to Fe^2+^ in a dentate bound (Figure 1). This makes the C4 of L-Ile in proximity to the Fe^2+^ (5.9 Å) and C2 of α-KG (4.7 Å). Such a distance may facilitate the attack of oxygen molecules (the diameter of oxygen molecule is about 3.5 Å) on C4 of L-Ile, where hydroxylation occurs [21,22]. Thus, the distance between Fe^2+^, C2 of α-KG and the fourth carbon of L-Ile (where hydroxylation occurs) may be crucial for the incorporation of oxygen molecules and oxidation reactions and important for substrate recognition and catalytic orientation.

**Figure 2:**
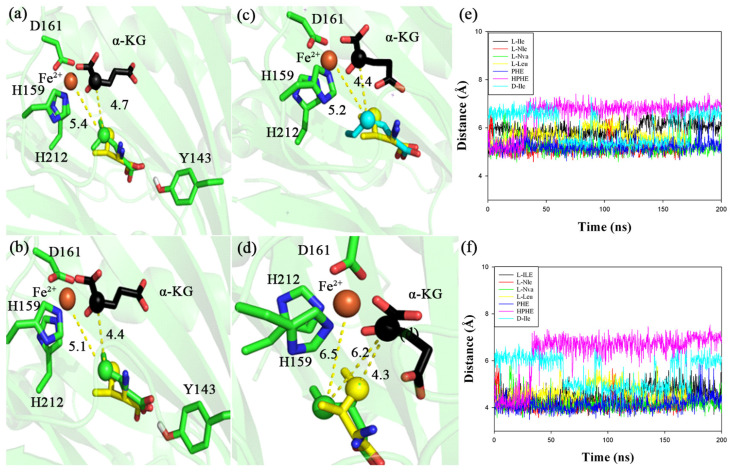
Molecular structure around active site of different substrates during molecular dynamics simulation at 200 ns. IDO substrates are L-leucine (L-Leu, (**a**), green), L-norvaline (L-Nva, (**b**), green), L-norleucine (L-Nle, (**c**), cyan), D-isoleucine (D-Ile, (**d**), green) and L-isoleucine (L-Ile, (**a**–**d**), yellow), respectively. Distance between C4 of different substrates and Fe^2+^ (**e**), C2 of α-KG (**f**) during IDO molecular dynamics simulation. L-Ile (black), L-Nle (red), L-Nva (green), L-Leu (yellow), PHE (blue), HPHE (purple), D-Ile (cyan). The unit of distance is Å.

IDO has a certain substrate spectrum for L-amino acids [17,18,19]. To explore this regularity, we chose L-aliphatic amino acids that can be catalyzed by IDO (although with lower catalytic efficiency), including L-Leu, L-Nva and L-Nle, and analyzed the reaction mechanism by MD simulations [17]. MD simulations showed that the closest distance to Fe^2+^ was located at C4 of the carbon chain (Fe^2+^ distances to L-Leu, L-Nva and L-Nle: 5.4, 5.1 and 5.2 Å, respectively) (Figure 2a–c). It is also interesting to note that the distance between C2 of α-KG and the C4 position of these L-aliphatic amino acids remains essentially around 4.4 Å (Figure 2), while it is as long as 6.2 Å in the D-Ile (L-Ile tautomer with a similar structure) which does not react with IDO (Figure 2d). Furthermore, MD simulations showed that the distance between Fe^2+^, C2 of α-KG and C4 position of D-Ile fluctuated greatly and was very unstable, whereas the other amino acids quickly reached a stable state (Figure 2e,f). This distance between Fe^2+^, C2 of α-KG and C4 of the L-aliphatic amino acid may be more favorable to IDO hydroxylated amino acid at the C4 position. Previous studies on Fe (II)/α-KG DOs suggest that α-KG binds reactive ferrous ions and displaces two water molecules to form an octahedral ligand complex [23,24,25]. During the reaction, one of the water molecules involved in the octahedral ligand complex formation is replaced by a molecule of oxygen, which, through oxidative decarboxylation, produces 1,4-butanedioic acid, CO_2_ and iron oxide [Fe(IV)=O], followed by high-valent iron interactions with the substrate to undergo hydroxylation [26,27,28,29]. In contrast, the distance between α-KG and the C4 position of the L-aliphatic amino acid may make the geometrical configuration of Fe-O more favorable to attack the C4 position of the amino acid for hydroxylation reactions through spatial crowding. This may be one reason why IDO has a favorable substrate spectrum for L-aliphatic amino acids.

### 2.2. Analysis of the Interaction between IDO and Aromatic Amino Acids

**Figure 3:**
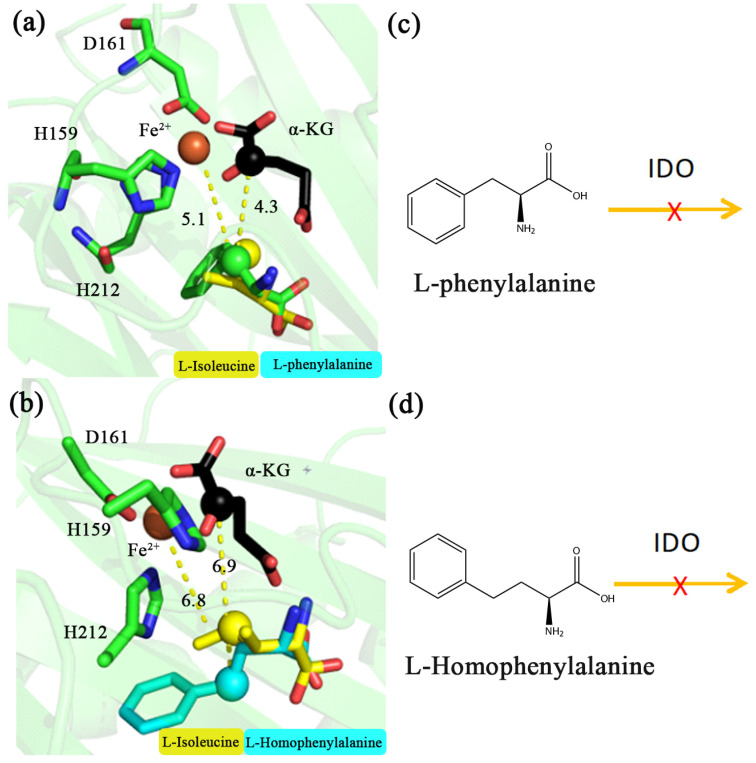
Molecular structure around active site of L-phenylalanine and L-homophenylalanine during molecular dynamics simulation at 200 ns. IDO substrates are L-phenylalanine (**a**), green, L-homophenylalanine (**b**), cyan and L-isoleucine (**a**,**b**), yellow, respectively. Pattern of L-phenylalanine (**c**) and L-homophenylalanine (**d**) that cannot be catalyzed by IDO. The unit of distance is Å.

Based on the above catalytic mechanism of IDO for L-aliphatic amino acids, MD simulations were carried out for aromatic amino acids (PHE and HPHE) to speculate whether IDO could react with them. MD simulations showed that the carbon chain orientation of PHE and HPHE was consistent with the above L-aliphatic amino acids. The distances between Fe^2+^ and the C4 position of PHE and HPHE were 5.1 and 6.8 Å, respectively (Figure 3a,b), and the distances between the C2 of α-KG and the C4 position of these amino acids were 4.3 and 6.9 Å, respectively (Figure 3a,b). Previous reaction patterns suggested that IDO recognition of the substrate requires hydroxylation occurs at methylene or methine carbon of the amino acid but not methyl [17]. Therefore, although C4 of PHE is in close proximity to Fe^2+^ and C2 of α-KG (5.1 and 4.3 Å, respectively), C4 of the carbon chain is a benzene ring, not a methylene or methine carton, and therefore presumably IDO cannot catalyze its hydroxylation. In contrast, C4 of HPHE is farther away from Fe^2+^ and C2 of α-KG (6.8 and 6.9 Å, respectively) and presumably cannot be hydroxylated by IDO. To further test this hypothesis, we purified the IDO protein and used IDO to catalyze the reaction with PHE and HPHE in vitro, showing no reaction between them, suggesting that IDO cannot catalyze PHE and HPHE (Figure 3c,d).

### 2.3. Structure-Guided Prediction of IDO Mutation Sites

**Figure 4:**
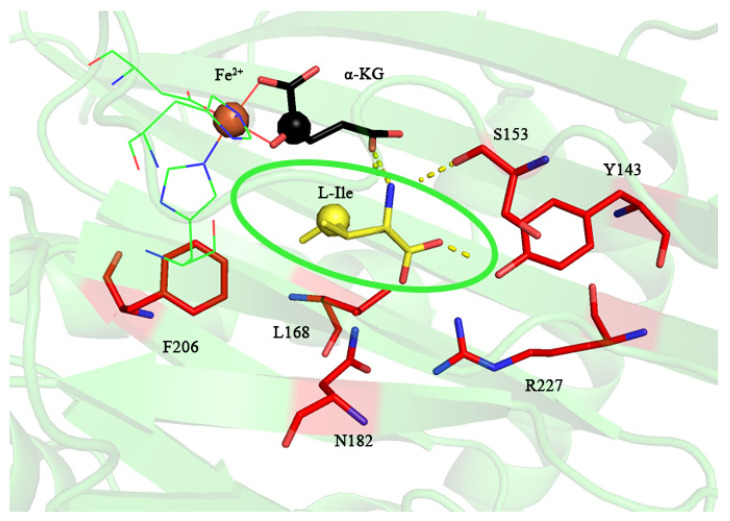
Display diagram in energy contribution of IDO key residues. The green elliptical line represents the substrate molecule L-Ile.

To extend the reaction of IDO toward aromatic amino acids, we used MD simulations for the selection of IDO mutation sites. There was an energy difference between the free and the bound states of the ligand and the receptor of a protein, which can be used to measure the affinity of their binding [30,31,32]. Analysis of the residue contribution using binding free energy calculations would be helpful to understanding the catalytic mechanism and thus to designing enzyme modification at the molecular level [32]. To further investigate the contribution of IDO residues to the reaction, the binding energies of the IDO, Fe^2+^, α-KG and L-Ile complex systems were simulated and calculated by molecular dynamics, with a binding energy of −34.11 kcal/mol for the IDO complex system, and the top ten residues contributing to the selectivity were labeled by decomposing their contribution to the binding energy (Table 1), among which the top ranked residues are mostly hydrophilic residues, indicating that hydrophilicity is a key factor affecting IDO catalysis. The residues with an absolute value of binding energy contribution greater than 1 kcal/mol were displayed at 200 ns, and it can be seen that these residues are distributed near the binding cavity substrate (Figure 4). Based on the residue contribution and polarity pockets (S153, P155, Y143 and R227), we selected Y143, S153 and R227, which contribute more than others, as the mutation sites.

### 2.4. Construction, Expression and Purification of IDO Mutant

**Figure 5:**
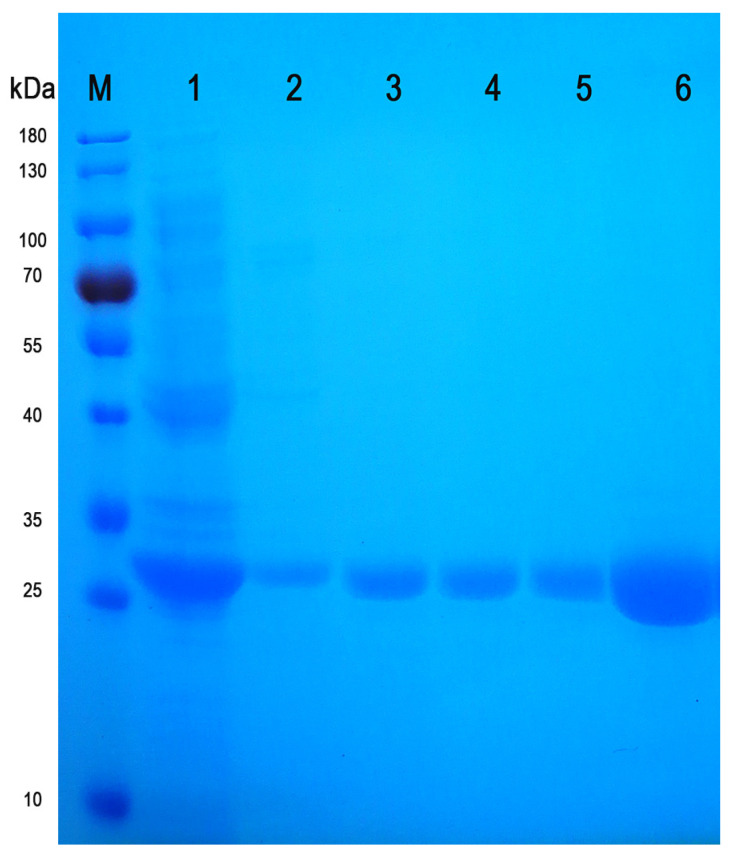
SDS-PAGE electrophoresis diagram of mutant IDO and IDO-WT. Protein marker (M), representative total protein expression (line 1), representative purified mutant IDO (lines 2–5), purified IDO-WT (line 6).

The single point saturation mutation of residues Y143, S153 and R227 were performed by gene synthesis, respectively, and 5′(NcoI), 5′UTR (AA) and 3′(SalI) were added at both ends of the sequence and cloned into the vector pET-28a (+). The constructed pET-28a (+)-*ido* mutant vector was confirmed by EcoRV and Xhol digestion and sequencing. Different IDO mutants were expressed in *E. coli* BL21 (DE3), and then the proteins were extracted and purified by ultrasound and a Ni column (Figure 5). The single point saturation mutation at residue Y143, S153 or R227 did not have an obvious influence on protein expression.

### 2.5. Enzymatic Activity Assay and Kinetic Characterization of IDO-WT and IDO Mutants 

We performed enzyme activity assays on IDO-WT and IDO mutants. The enzyme activity results showed that IDO-WT had high enzyme activity (0.55 ± 0.15 μmol/min/mg) on the optimal substrate L-Ile, but no enzyme activity on PHE and HPHE was detected. This indicated that IDO could not catalyze PHE and HPHE. We unexpectedly found that mutants Y143D, Y143I and S153A showed catalytic activity of PHE, and their enzyme activities were 0.056 ± 0.008, 0.025 ± 0.003 and 0.012 ± 0.002 μmol/min/mg (Table 2), respectively. We also found that the mutants Y143I, S153A, S153Q and S153Y were active toward HPHE with enzymatic activities of 0.014 ± 0.002, 0.035 ± 0.005, 0.063 ± 0.003 and 0.028 ± 0.004 μmol/min/mg (Table 2), respectively. To further investigate the enzyme’s ability to bind the substrate and catalyze the reaction rate, the enzyme kinetic parameters were examined, and the results are shown in Table 3. For PHE, among all mutants, the *k_cat_/K_m_* value of IDO-Y143I was the highest at 2.00 ± 0.01 s^−1^mM^−1^, indicating that the IDO-Y143I had the highest catalytic efficiency. For HPHE, among the various mutants, the *k_cat_/K_m_* value of IDO-S153Q was the highest at 3.50 ± 0.10 s^−1^mM^−1^, indicating that IDO-S153Q had the highest catalytic efficiency.

To identify whether the product of the catalytic reaction was hydroxylated, LC-MS was performed. The results obtained from LC-MS analyses showed the presence of mass spectrometric peaks consistent with the molecular weight of the hydroxylated products, thereby confirming the formation of 2-amino-3-hydroxy-3-phenylpropanoic acid after hydroxylation of PHE catalyzed by mutant Y143D, Y143I and S153A (Figures S1–S3). Our findings verified the potential of IDO mutants to catalyze the hydroxylation of PHE. 

These results indicated that the modification of the IDO molecule realizes its possibility to catalyze aromatic amino acids, although their catalytic activity is low. This will provide an idea to further improve the activity of IDO catalyzed aromatic amino acids.

### 2.6. Molecular Docking Analysis 

**Figure 6:**
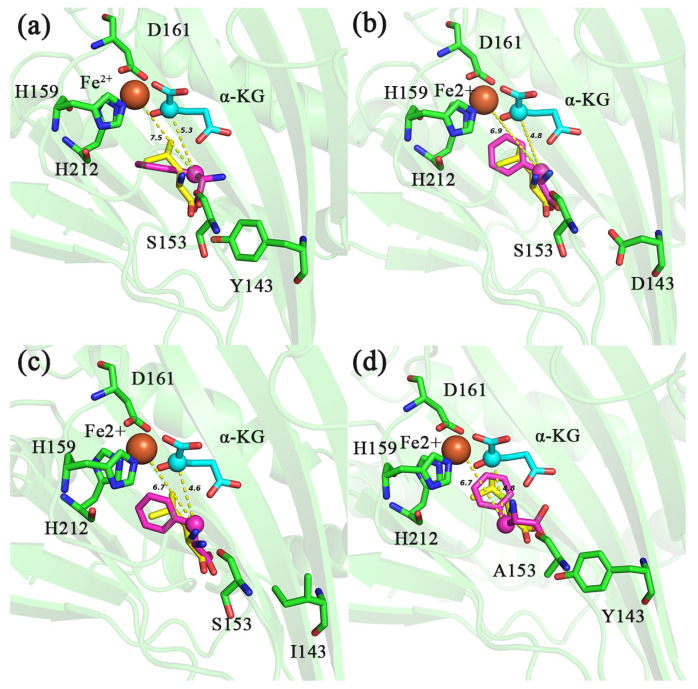
Molecular docking diagram of different IDO mutants (Y143D-IDO: (**b**); Y143I-IDO: (**c**); S153A-IDO: (**d**)) and IDO-WT (**a**) with PHE and L-Ile. Distance between C2 of α-KG, Fe^2+^ and C4 of different substrates is indicated by yellow dashed lines. PHE (purple), L-Ile (yellow). The unit of distance is Å.

The previous analysis has shown that because the C4 position of the PHE side chain is a benzene ring but not methylene or methine carbon, IDO cannot hydroxylate with it, although the C4 of the carbon chain is close to Fe^2+^ and C2 of α-KG [17]. However, these mutants showed the activity of catalyzing PHE, and we speculated that the methylene at position C3 was hydroxylated [17]. Molecular docking results showed that the distance between C3 of PHE and Fe^2+^ in mutants Y143D, Y143I and S153A was 6.9, 6.7 and 6.7 Å (Figure 6b–d), respectively, and the distances to C2 of α-KG was 4.8, 4.6 and 4.8 Å (Figure 6b–d), respectively, which was closer than that of *d* (Fe^2+^-PHE_C3_) (7.5 Å), *d* (α-KG_C2_-PHE_C3_) (5.3 Å) in IDO-WT (Figure 6a). Moreover, the binding free energies of these mutants to PHE were decreased (−6.2, −6.5, −6.4 and −6.5 kcal/mol for IDO-WT, Y143D, Y143I and S153A, respectively) (Table 4), which may favor the hydroxylation of Fe-O attacking C3 of PHE to produce the non-essential amino acid 2-amino-3-hydroxy-3-phenylpropanoic acid. Of course, this does not exclude the possibility of the positive mutant also acting on the benzene ring [33,34,35,36]. In the future, we should accurately detect the reaction site in the tested amino acid substrate.

**Figure 7:**
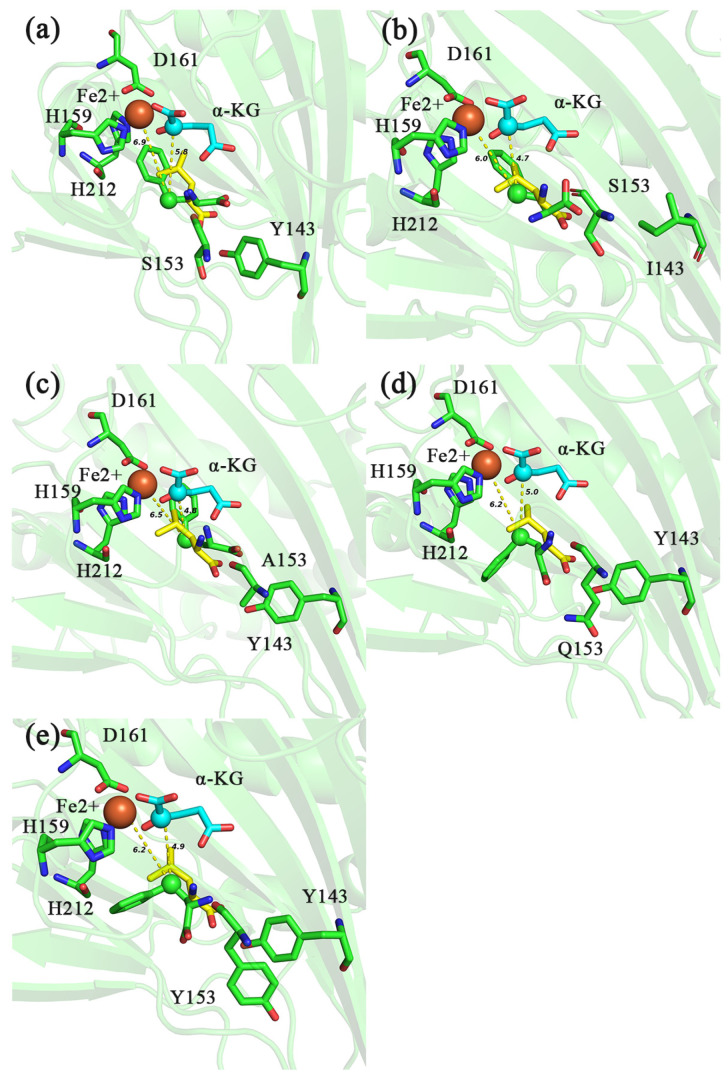
Molecular docking diagram of different IDO mutants (Y143I-IDO: (**b**); S153A-IDO: (**c**); S153Q-IDO: (**d**); S153Y-IDO: (**e**)) and IDO-WT (**a**) with HPHE and L-Ile. Distance between C2 of α-KG, Fe^2+^ and C4 of different substrates is indicated by yellow dashed lines. HPHE (green), L-Ile (yellow). The unit of distance is Å.

The molecular docking results showed that the distance of C4 of HPHE from Fe^2+^ and C2 of α-KG in IDO-WT was 6.9 and 5.8 Å, respectively (Figure 7a), while the distance of C4 of HPHE from Fe^2+^ in mutants Y143I, S153A, S153Q and S153Y were 6.0, 6.5, 6.2 and 6.2 Å, respectively (Figure 7b–e), and the distances to C2 of α-KG were 4.7, 4.8, 5.0 and 4.9 Å (Figure 7b–e), respectively. This indicated that C4 of HPHE in the mutant was closer to Fe^2+^ and C2 of α-KG than that in the wild type, which would be more favorable for C4 of HPHE to be attacked. Moreover, the binding free energy analysis showed that the binding free energies of Y143I, S153A, S153Q and S153Y to HPHE (−6.6, −6.5, −6.4 and −6.5 kcal/mol) were slightly lower compared to IDO-WT (−6.3 kcal/mol) (Table 4). Taken together, these site mutations may be more favorable for hydroxylation on C4 of PHE.

However, the saturated mutant at residue R227 showed no catalytic activity for the aromatic amino acids (PHE and HPHE) (Table 2). This may be due to the influence of R227 on the conformation of the amino group of the amino acid, thus affecting the orientation of the substrate, which cannot keep an appropriate distance between the C3 or C4 of PHE and HPHE chains from Fe^2+^ and C2 of α-KG.

## 3. Materials and Methods

### 3.1. Materials

The bacterial strains and plasmids used in this study are listed in Table 5. *E. coli* DH5α was used as a cloning host. *E. coli* BL21(DE3) was used for expression of ido and mutant genes. The chemicals, including α-KG, L-Ile, L-Leu, L-Nva, L-Nle, D-Ile, PHE, HPHE, NaH_2_PO_4_, FeSO₄·7H₂O, NaCl, kanamycin, Isopropyl β-D-thiogalactoside (IPTG), imidazole and LB medium, were purchased from Sigma-Aldrich.

### 3.2. Mutagenesis Vector Construction

For saturation mutagenesis at each site, the *ido* mutant genes were designed by replacing the codon encoding the predicted residue with those encoding other 19 residues separately. A total of 57 *ido* mutant genes were synthesized by Suzhou GENEWIZ Biotechnology Co., Ltd. (Suzhou, China), with added 5′(NcoI), 5′UTR ([AA]) and 3′(SalI). The gene was cloned into expression vector pET-28a (+) (Kanamycin) by 5′ NcoI and 3′ SalI to construct pET-28a (+)-*ido mutant*, and then the plasmid was transformed into *E. coli* BL21 (DE3) for subsequent expression of the target protein [37].

### 3.3. Protein Expression and Purification

The plasmid, pET-28a (+)-*ido* involving wild-type *ido* gene or pET-28a (+)-*ido mutant* involving *ido* mutant gene, was transformed into *E. coli* BL21 (DE3) and smeared on an LB plate containing 50 μg/mL kanamycin. Single colonies were selected and identified by enzyme digestion. The single colonies of the recombinant strain were inoculated in 5 mL LB medium containing 50 μg/mL kanamycin and cultured overnight at 37 °C with 200 r/min shaking. The 5 mL seed liquid was transferred to 500 mL liquid medium containing 50 μg/mL kanamycin in LB medium. At 37 °C, the medium was shaken at 200 r/min and cultured to OD_600_ of about 0.6–0.8. IPTG with a final concentration of 1 mM was added to the medium and induced and cultured overnight at 25 °C and 200 r/min [37].

The precipitate was collected by centrifugation at 4000× *g* for 20 min and suspended in a lysis solution (50 mM NaH_2_PO_4_, 300 mM NaCl, pH 8.0). The cells resuspended in the lysate above were sonicated in an ice bath to break the bacteria. Sonication conditions: 360 w, 2 s of work, 3 s of interval, 15 min and 4 °C. The supernatant was collected by 12,000× *g*/min centrifugation for 20 min. The supernatant was filtered through a 0.22 μm membrane, and the crude enzyme solution was used for further purification.

The crude enzyme solution was then purified by applying a Ni-Charged Resin FF column (GenScript, Nanjing, China). Purification conditions: Wash buffer 1 (50 mM NaH_2_PO_4_, 300 mM NaCl, 150 mM imidazole, pH 8.0) and wash buffer 2 (50 mM NaH_2_PO_4_, 300 mM NaCl, 250 mM imidazole, pH 8.0) were used to elute non-target proteins. The target proteins were finally eluted with elution buffer (50 mM NaH_2_PO_4_, 300 mM NaCl, 500 mM imidazole, pH 8.0). A 12% SDS-PAGE was performed to determine the size of the target protein molecule and the degree of purification [38].

### 3.4. Enzyme Activity Determination

In this work, the enzyme activity of IDO and its variants was assayed by measuring the yield of succinic acid, which was generated accompanying the IDO-catalyzed hydroxylation. One unit of enzyme activity was defined as the amount of enzyme required to produce 1 μmol succinate per minute in a standard enzyme assay system. The catalytic reaction system configuration was as follows: 10 mM substrate, 10 mM α-KG, 0.5 mM FeSO_4_·7H_2_O, 10 mM ascorbic acid, 50 mM Bis-Tris (pH 6.0) and 0.2 mg/mL purified IDO or IDO mutants in 1 mL; reaction conditions: 25 °C, 10 min. The supernatant was collected by centrifugation at 4000× *g* for 10 min at 4 °C. The supernatant was assayed using the Succinic Acid Assay Kit (MAK184, Sigma-Aldrich, Inc., Shanghai, China). All assays were performed in triplicate.

### 3.5. Determination of Kinetic Parameters

The kinetic parameters were determined using 1 mL of the assay mixture. For PHE and HPHE, the standard conditions for the hydroxylation reaction and the activity assay with the Succinic Acid Assay method were used, and the concentration of PHE and HPHE was 0.01–5 mM. The assays were carried out in triplicate, and the Michaelis–Menten model was fitted using GraphPad Prism 9 (GraphPad, Inc., San Diego, CA, USA).

### 3.6. Analysis of the Catalytic Product 

The products of the amino acid hydroxylation were analyzed using the Waters ACQUITY UPLC-MS/MS system. A Waters ACQUITY UPLC HSS C18 column (1.8 μm) was used. Gradient elution was performed for mobile phase A consisting of 20 mM NH4Ac-HAc buffer (pH 4.2) and mobile phase B consisting of acetonitrile. The flow rate was 200 μL/min, the column temperature was 25 °C, and the sample size was 5 μL.

### 3.7. Molecular Docking and Molecular Dynamics (MD) Simulation Analysis

The IDO monomer A (PDB: 6lnh, Chain A) containing an Fe ion was used as the initial structure of the enzyme, and the Fe ion was used as the reference site of the active center. α-KG and amino acids (L-Ile, L-leu, L-Nva, L-Nle, D-Ile, PHE and HPHE) were carefully aligned to the active site by AutoDock Vina. The protein atom was described by an AMBER18 ff14SB force field, and the substrate and 2OG were parameterized by the general AMBER force field (GAFF) [39,40]. The metal central bonding model was applied to this system. The whole modeling process was completed under the guidance of the Metal Center Parameter Builder (MCPB) program, which included the calculation of force constants, geometric optimization of active center residues and restrained electrostatic potential charge (RESP charge) using Gaussian09 [41]. After obtaining the force field parameters, AMBER further minimized the system to obtain the correct molecular structure. At 200 ns, the C1-N1-C4 atoms of other amino acid substrates were aligned with the L-Ile model structure, and a good docking structure was obtained.

The systems were then solvated in a rectangular box of TIP3P water and were neutralize with sodium ions. The total number of systems was about 32,000. The positions of water were then optimized with 800 steps of the steepest descent (SD) method and 9200 steps of the conjugate gradient (CD) method followed by 1800 steps of steepest descent (SD) method and 38,200 steps of the conjugate gradient (CD) method to the entire system. After that, the systems were gradually heated from 0 to 300 K over 50 ps in the NVT ensemble. Then, the systems were equilibrated for 75 ps under 1 atm of pressure. Finally, a molecular simulation of 200 ns was performed for each system, during which the Verlet algorithm with a time step of 2 fs was employed and the SHAKE algorithm adopted for the constraint of bond stretching of those covalent bonds involving hydrogen atoms [42,43].

To understand the interaction of IDO mutants with the substrates PHE and HPHE, we selected IDO-WT and mutants to dock PHE and HPHE, respectively. Molecular docking was performed by AutoDock Vina, based on the substrate L-Ile of the original IDO and its surrounding residues as the active pocket. At the end of docking, the scored optimal conformation was selected to merge with the protein and saved as a complex. The small molecule-receptor binding site and minimum binding energy data were obtained by Pymol and AutoDock Vina, respectively.

## 4. Conclusions

In this study, the regularity of the wider substrate spectrum of IDO was explored using our previously obtained IDO structure in combination with molecular docking and MD simulations, i.e., the distance of C4 on the amino acid chain to Fe^2+^ and C2 of α-KG may be critical. The selection of mutation sites for single point saturation mutagenesis based on polarity pockets and residue free energy contribution revealed mutations in sites Y143 and S153 that expanded the substrate spectrum of IDO toward aromatic amino acids, despite their low catalytic activity. In the future, the Y143 and S153 sites can be mutated simultaneously with IDO sites around the benzene ring to further improve their catalytic activity for aromatic side-chain amino acids, thus expanding the reaction of IDO with aromatic amino acids to enhance its application performance.

## Figures and Tables

**Table 1:** Energy contribution of IDO key residues.

Residue	Residue Number	Binding Energy Decomposition (kcal/mol)
H	212	−0.59
A	213	−0.791
P	155	−0.852
L	168	−1.188
F	206	−1.61
V	214	−1.66
Y	143	−1.73
S	153	−2.414
N	182	−2.929
R	227	−4.06

**Table 2:** Specific activity of IDO mutants.

Enzyme	Specific Activity (μmol/min/mg)		
	L-Ile	PHE	HPHE
IDO-WT	0.550 ± 0.150	N	N
IDO-Y143D	N	0.056 ± 0.008	N
IDO-Y143I	N	0.025 ± 0.003	0.014 ± 0.002
IDO-S153A	N	0.012 ± 0.002	0.035 ± 0.005
IDO-S153Q	N	N	0.063 ± 0.003
IDO-S153Y	0.131 ± 0.040	N	0.028 ± 0.004

Note: WT: wild type; N, not detected.

**Table 3:** Kinetic parameters of IDO-WT and mutants.

Enzyme	Substrate	*K_m_* (mM)	*V_max_* (μmol·min^−1^·mg^−1^)	*K_cat_* (s^−1^)	*K_cat_*/*K_m_* (s^−1^mM^−1^)
IDO-WT	PHE	N	N	N	N
	HPHE	N	N	N	N
IDO-Y143D	PHE	0.04 ± 0.00	0.15 ± 0.02	0.08 ± 0.00	2.00 ± 0.01
	HPHE	N	N	N	N
IDO-Y143I	PHE	0.03 ± 0.00	0.10 ± 0.01	0.04 ± 0.00	1.33 ± 0.04
	HPHE	0.02 ± 0.00	0.08 ± 0.01	0.04 ± 0.00	2.00 ± 0.08
IDO-S153A	PHE	0.02 ± 0.00	0.05 ± 0.01	0.03 ± 0.00	1.50 ± 0.05
	HPHE	0.02 ± 0.00	0.09 ± 0.02	0.05 ± 0.00	2.50 ± 0.04
IDO-S153Q	PHE	N	N	N	N
	HPHE	0.04 ± 0.01	0.21 ± 0.04	0.14 ± 0.01	3.50 ± 0.10
IDO-S153Y	PHE	N	N	N	N
	HPHE	0.02 ± 0.00	0.08 ± 0.01	0.03 ± 0.00	1.50 ± 0.04

Note: WT: wild type; N, not detected.

**Table 4:** Receptor–ligand affinity.

Receptor	L-Phenylalanine Affinity (kcal/mol)	L-Homophenylalanine Affinity (kcal/mol)
IDO	−6.2	−6.3
Y143D-IDO	−6.5	−6.6
Y143I-IDO	−6.4	−6.5
S153A-IDO	−6.5	−6.3
S153Q-IDO	−6.3	−6.4
S153Y-IDO	−6.1	−6.5

**Table 5:** Strains and plasmids.

Strain or Plasmid	Description	Source or Reference
*E. coli* DH5α	F^−^ *endA1 glnV44 thi-1 recA1 relA1 gyrA96 deoR nupG Φ80dlacZΔM15, Δ(lacZYA-argF)U169, hsdR17(rK^−^ mK^+^)*, λ^–^	TaKaRa
*E. coli* BL21(DE3)	F^−^ *ompT hsdS_B_(r_B_ ^−^ m_B_ ^−^) gal dcm* (DE3)	Novagen/Millipore
pET-28a (+)-*ido*	*kan, ido* (*Bacillus thuringiensis*)	Our previous study
pET-28a (+)-*ido mutants*	Single point saturation mutation Y143, S153 and R227	This study

## Data Availability

The experimental data provided in this work are available in articles.

## Supplementary Materials

The following supporting information can be downloaded at: https://www.mdpi.com/article/10.3390/molecules28093750/s1, Figure S1: LC-MS analysis of products catalyzed by Y143D with substrate PHE; Figure S2: LC-MS analysis of products catalyzed by Y143I with substrate PHE; Figure S3: LC-MS analysis of products catalyzed by S153A with substrate PHE.

## Sample Availability

IDO mutants are available from the authors.
