# Supplementary material for: Semi-Rational Design of L-Isoleucine Dioxygenase Generated Its Activity for Aromatic Amino Acid Hydroxylation
Source: Molecules. 2023 Apr 27;28(9):3750. doi: 10.3390/molecules28093750 (PMC10180240; doi:10.3390/molecules28093750)
Supplement: Supplementary file 1 [file molecules-28-03750-s001.zip › molecules-2307388-supplementary.pdf]

Supplemantary Information

Semi-Rational Design of L-Isoleucine Dioxygenase Generated Its Activity for Aromatic Amino Acid Hydroxylation

Jianhong An [1,2,3], Jiaojiao Guan [1] and Yao Nie [1,2,*]

1 School of Biotechnology and Key Laboratory of Industrial Biotechnology, Ministry of Education, Jiangnan University, 1800 Lihu Road, Wuxi 214122, China; ajhbio1978@126.com (J.A.); 15906191696@139.com (J.G.)
2 International Joint Research Laboratory for Brewing Microbiology and Applied Enzymology, Jiangnan University, 1800 Lihu Road, Wuxi 214122, China
3 Eye Hospital, Wenzhou Medical University, 270 Xueyuan Road, Wenzhou 325000, China
* Correspondence: ynie@jiangnan.edu.cn; Tel.: +86-510-85197760; Fax: +86-510-85918201

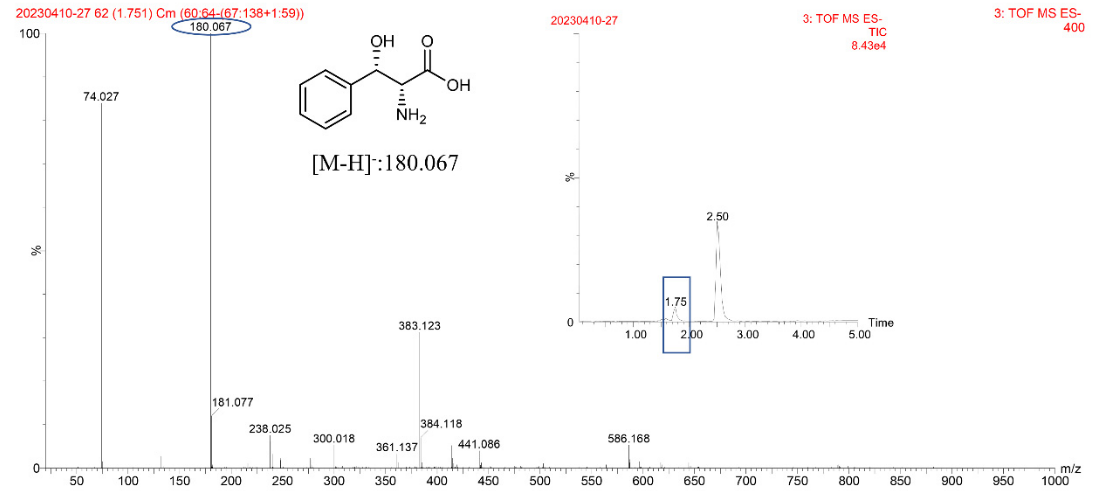

Figure S1. LC-MS analysis of products catalyzed by Y143D with substrate PHE.

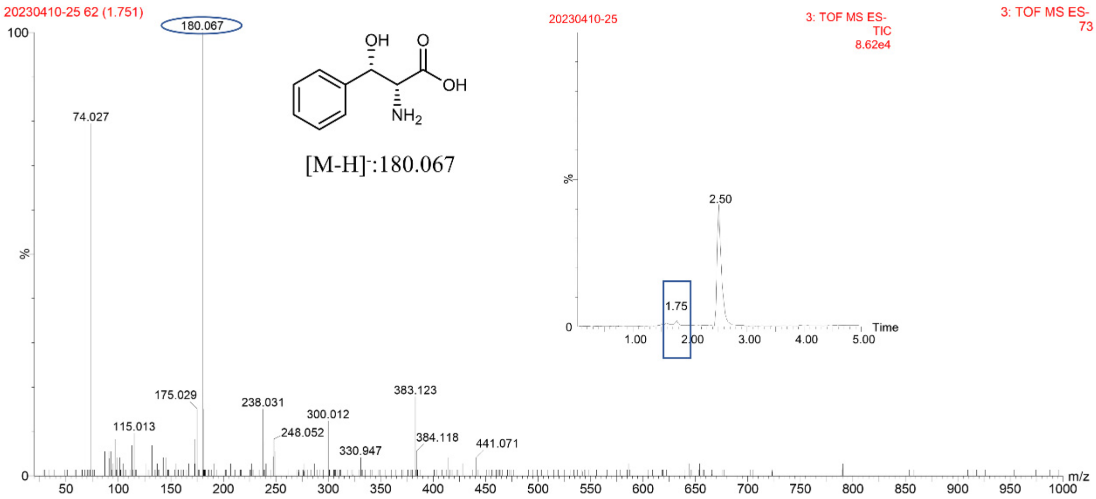

Figure S2. LC-MS analysis of products catalyzed by Y143I with substrate PHE.

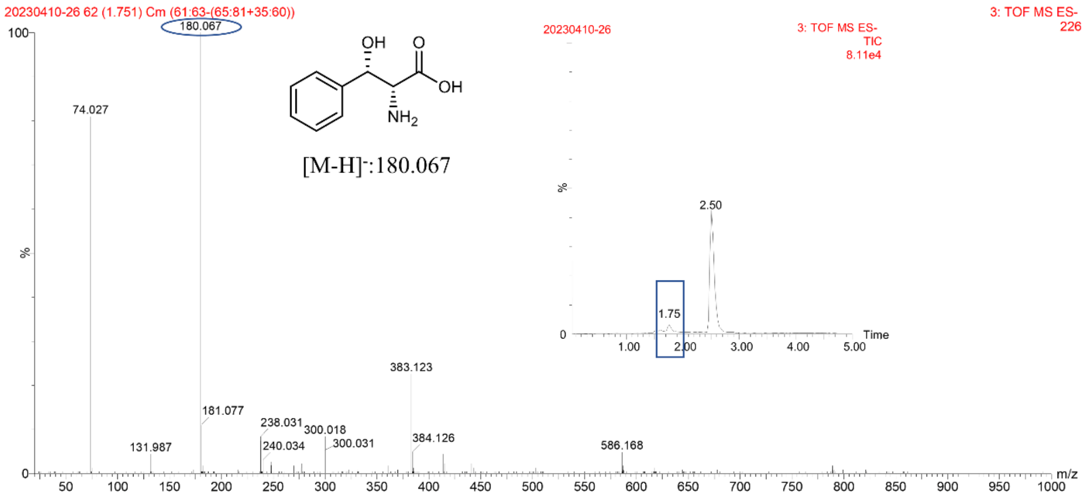

Figure S3. LC-MS analysis of products catalyzed by S153A with substrate PHE.
